# Solvability of some partial functional integrodifferential equations with finite delay and optimal controls in Banach spaces 

**DOI:** 10.1186/s40064-016-2896-8

**Published:** 2016-08-05

**Authors:** Khalil Ezzinbi, Patrice Ndambomve

**Affiliations:** 1 Département de Mathématiques, Faculté des Sciences Semlalia, Université Cadi Ayyad, B.P. 2390, Marrakech, Morocco; 2 Department of Mathematics, Faculty of Science, University of Buea, P.O. Box 63, Buea, Cameroon

**Keywords:** Partial functional integrodifferential equations, Finite delay, Resolvent operator, Solvability, Mild solutions, Optimal controls, 93B05, 45K05, 47H08, 47H10, 34K37

## Abstract

In this work, we consider the control system governed by some partial functional integrodifferential equations with finite delay in Banach spaces. We assume that the undelayed part admits a resolvent operator in the sense of Grimmer. Firstly, some suitable conditions are established to guarantee the existence and uniqueness of mild solutions for a broad class of partial functional integrodifferential infinite dimensional control systems. Secondly, it is proved that, under generally mild conditions of cost functional, the associated Lagrange problem has an optimal solution, and that for each optimal solution there is a minimizing sequence of the problem that converges to the optimal solution with respect to the trajectory, the control, and the functional in appropriate topologies. Our results extend and complement many other important results in the literature. Finally, a concrete example of application is given to illustrate the effectiveness of our main results.

## Background

The aim of this work is to study the existence of mild solutions and the optimal controls of some systems that arise in the analysis of heat conduction in materials with memory (Grimmer 1983), and viscoelasticity and take the form of the following partial functional integrodifferential equation with finite delay in a Banach space $(X,\ \Vert \cdot \Vert )$.

1 $$\begin{aligned} \left\{ \begin{array}{l} x'(t)=Ax(t) + {\int _0^tB(t-s)x(s)ds} + f(t,x_t)+C(t)u(t)\quad {\text {for}}\ t\in I=[0,b] \\ x_0 = \varphi \in {\mathcal {C}}={\mathcal {C}}([-r,0];X), \end{array}\right. \end{aligned}$$

where $f{:}\, I\times {\mathcal {C}}\rightarrow X$ is a function satisfying some conditions; $A:{\mathcal {D}}(A)\rightarrow X$ is the infinitesimal generator of a *C*_0_-semigroup $\left( T(t)\right) _{t\ge 0}$ on a separable reflexive Banach space *X*; for *t* ≥ 0, *B*(*t*) is a closed linear operator with domain ${\mathcal {D}}(B(t))\supset {\mathcal {D}}(A)$. The control *u*(*t*) takes values from another separable reflexive Banach space *U*. The operator *C*(*t*) belongs to ${\mathcal {L}}(U,X)$, the Banach space of bounded linear operators from *U* into *X*, and ${\mathcal {C}}([-r,0],X)$ denotes the Banach space of continuous functions $\varphi{:}\, [-r,0]\rightarrow X$ with supremum norm $\Vert \varphi \Vert =\sup \nolimits _{\theta \in [-r,0]}\Vert \varphi (\theta )\Vert$, *x*_*t*_ denotes the history function of ${\mathcal {C}}$ defined by

$$x_t(\theta )=x(t+\theta )\quad {\text {for}} \ \ \theta \in [-r,0].$$

In many areas of applications such as engineering, electronics, fluid dynamics, physical sciences, etc..., integrodifferential equations appear and have received considerable attention during the last decades. In Grimmer (1983), Grimmer has proved the existence and uniqueness of resolvent operators for these integrodifferential equations that give the variation of parameter formula for the solution. In recent years, much work has been done on the existence and regularity of solutions of nonlinear integrodifferential equations with finite delay by many authors by applying the resolvent operator theory, for integral equations see e.g., Ezzinbi et al. (2009) and the references therein. Problems of existence of optimal controls for nonlinear differential equations have been studied extensively by many authors under various hypotheses (see e.g., Boyarsky 1976; Flytzanis and Papageorgiou 1991; Hongwei 2003; Jakszto and Skowron 2003; Noussair et al. 1981; Papageorgiou 1987), but little is known and done about the existence of optimal controls for integrodifferential equations using the resolvent operator theory.

Wang and Zhou (2011) discussed the optimal controls of a Lagrange problem for the following fractional evolution equations:

$$\begin{aligned} \left\{ \begin{array}{l} D^{q}x(t)=-Ax(t)\ + f(t,x(t))+C(t)u(t)\quad {\text {for}}\ \ t\in [0,b]\\ x(0) = x_0\in X, \end{array} \right. \end{aligned}$$

where $D^{q}$ denotes the Caputo fractional derivative of order $q\in (0,1)$ and $-A:{\mathcal {D}}(A)\rightarrow X$ is the infinitesimal generator of a compact analytic semigroup of uniformly bounded linear operators.

In Li and Liu (2015), the authors studied the existence of mild solutions and the optimal controls of a Lagrange problem for the following impulsive fractional semilinear differential equations,

$$\begin{aligned} \left\{ \begin{array}{l} ^CD^{\alpha }_tx(t)=Ax(t)\ + f\left( t,x(t), \int _0^tg(t,s,x(s))ds\right) +C(t)u(t)\quad {\text {for}}\ t\in [0,b],\quad t\ne t_k\\ \Delta x(t_k)=I_k(x(t_k^{-})),\quad k=1,2,\ldots , m\\ x(0) = x_0\in X, \end{array} \right. \end{aligned}$$

where $^CD^{\alpha }_t$ denotes the Caputo fractional derivative of order $\alpha \in (0,1]$ with lower limit zero and $A:{\mathcal {D}}(A)\rightarrow X$ is the infinitesimal generator of a *C*_0_-semigroup. They used the techniques of a priori estimation.

In Pan et al. (2014), the authors considered the following semilinear control systems with Riemann–Liouville fractional derivatives:

$$\begin{aligned} \left\{ \begin{array}{l} ^LD^{\alpha }_tx(t)=Ax(t) + f(t,x(t))+Cu(t)\quad {\text {for}}\ t\in (0,b]\\ I^{1-\alpha }_{0^{+}}x(t)|_{t=0} = x_0\in X, \end{array} \right. \end{aligned}$$

where $0<\alpha <1,\ ^LD^{\alpha }_t$ denotes the Riemann–Liouville fractional derivative of order $\alpha$ with the lower limit zero. and $A:{\mathcal {D}}(A)\rightarrow X$ is the infinitesimal generator of a C$_0$-semigroup.

In Wang et al. (2012), the authors considered the following fractional integrodifferential equation with infinite delay in Banach spaces

$$\begin{aligned} \left\{ \begin{array}{l} ^CD^q_tx(t)=Ax(t) + f\left( t,\,x_t, \int _0^tg(t,\,s,x_s)ds\right) \ +C(t)u(t)\quad {\text {for}}\ t\in I=[0,b] \\ x_0 = \varphi \in {\mathcal {B}}, \end{array} \right. \end{aligned}$$

where $^CD^q_t$ denotes the Caputo fractional derivative of order $q\in (0,1)$. Using the using the techniques of a priori estimation and extension of step by steps, they studied the existence and continuous dependence of mild solutions and the optimal controls of the associated Lagrange problem.

Zhou (2014) considered a controlled stochastic delay partial differential equation with Neumann boundary conditions and studied the optimal control problem by means of the associated backward stochastic differential equations. In Motta and Rampazzo (2013), the authors discussed the assymptotic controllability and the optimal control of some control system where the state approaches asymptotically a target, while paying an integral cost with a nonnegative Lagrangian. Wang and Zhou (2011) discussed the optimal controls of a Lagrange problem for fractional evolution equations. In Wei et al. (2006), the authors studied the optimal controls for nonlinear impulsive integrodifferential equations of mixed type on Banach spaces. In Li and Liu (2015), the authors studied the existence of mild solutions and the optimal controls of a Lagrange problem for some impulsive fractional semilinear differential equations, using the techniques of a priori estimation.

Motivated by these works, we investigate the solvability and the existence of optimal controls of a Lagrange problem for Eq. (1), which is a more general class than those studied by the authors mentioned above. Using the techniques of a priori estimation of mild solutions and without any compactness assumptions made, the existence and uniqueness of mild solutions is obtained using the theory of resolvent operator for integral equations. Furthermore, to the best of our knowledge, the optimal controls for partial functional integrodifferential Eq. (1) with finite delay are untreated in the literature, and this fact motivates us to extend the existing ones and make new development of the present work on this issue.

## A model in heat conduction in materials with memory

As a motivation for the problem studied in this work, we consider a heat flow in a rigid body $\Omega$ of a material with memory. Let $w(t,\xi ),\ e(t,\xi ),\ q(t,\xi )$ and $s(t,\xi )$ denote respectively the temperature, the internal energy, the heat flux, and the external heat supply at time *t* and position $\xi$. The balance law for the heat transfer is given by:

2 $$e_t(t,\xi )+{\text {div}}\,q(t,\xi )= s(t,\xi )$$

and the physical properties of the body suggest the dependence of *e* and *q* on *w* and $\nabla w$, respectively. For instance assuming the Fourier Law i.e.,

3 $$e(t,\xi )=c_1w(t,\xi )$$

4 $$q(t,\xi )=-c_2\nabla w(t,\xi ),$$

where *c*_1_, *c*_2_ are positive constants, one deduces from (2) the classical heat equation

5 $$w_t(t,\xi )=c\Delta w(t,\xi )+g(t,\xi )$$

with $c=c_1^{-1}c_2$ and $g(t,\xi )=c_1^{-1}s(t,\xi )$. In many materials the assumptions (3), (4) are not justified because they take no account of the memory effects: several models have been proposed to overcome this difficulty, see e.g. Dafermos and Nohelj (1979), Gurtin and Pipkin (1968), Sinestrari (1987): one of them consists in substituting (4) with

6 $$q(t,\xi )=-c_2\nabla w(t,\xi )-\int _{-\infty }^th(t-s)\nabla w(s,\xi )ds.$$

Taking for simplicity $c_1=c_2=1$, we get from (2), (3) and (6)

7 $$w_t(t,\xi )=\Delta w(t,\xi )+ \int _{-\infty }^th(t-s)\Delta w(s,\xi )ds+s(t,\xi ).$$

If we assume that the thermal history *w* of the body $\Omega$ is known up to *t* = 0, and the temperature of the boundary $\partial \Omega$ of $\Omega$ is constant (=0) for all *t*, we are led to the following system:

8 $$\begin{aligned} \left\{ \begin{array}{l} w_t(t,\xi )=\Delta w(t,\xi )+ \int _{0}^{t}h(t-s)\Delta w(s,\xi )ds+g(t,\xi )\quad {\text {for}}\ (t,\xi )\in [0,b]\times \Omega \\ w(t,\xi )=0,\quad (t,\xi )\in [0,b]\times \partial \Omega , \end{array} \right. \end{aligned}$$

where *b* > 0 is arbitrarily fixed. If we prescribe *h* (in addition to *f*) then (8) is a Cauchy-Dirichlet problem for an integrodifferential equation in the unknown *w*, which has been studied by several authors in the last decades, see e.g., Grimmer and Pritchard (1983), Grimmer and Kappelf (1984), Lunardi and Sinestrari (1986) and references therein.

Now, if we consider that the thermal history of the body $\Omega$ is known from the time $t-r$ (for some *r* > 0) up to the present time *t*, the temperature of the boundary $\partial \Omega$ of $\Omega$ is constant (=0) for all *t*, and the external heat supply depends on the this thermal history of the body, then, system (8) becomes the following integrodifferential equation with finite delay:

9 $$\begin{aligned} \left\{ \begin{array}{l} w_t(t,\xi )=\Delta w(t,\xi )+ \int _{0}^{t}h(t-s)\Delta w(s,\xi )ds+g(t,w(t-r,\xi )),\\ \qquad\qquad\quad (t,\xi )\in [0,b]\times \Omega \\ w(t,\xi )=\psi (t,\xi )\quad {\text {for}}\ t\in [-r,0]\ {\text {and}}\ x\in \overline{\Omega }\\ \end{array}\right. \end{aligned}$$

where $\psi$ is a given initial function and *r* is a positive number. Let $\lambda (t)\beta (t,\xi )$ denote the heating intensity, added to the system to control and regulate the heat supply. Then system (9) becomes

10 $$\begin{aligned} \left\{ \begin{array}{ll} w_t(t,\xi )=\Delta w(t,\xi )+ \int _{0}^{t}h(t-s)\Delta w(s,\xi )ds+g(t,w(t-r,\xi ))+\lambda (t)\beta (t,\xi ),\\ \qquad\qquad (t,\xi )\in [0,b]\times \Omega \\ w(t,\xi )=\psi (t,\xi )\ \ {\text {for}}\ t\in [-r,0]\ {\text {and}}\ x\in \overline{\Omega }\\ \end{array} \right. \end{aligned}$$

Now define

$$\begin{aligned} x(t)(\xi )&= w(t,\xi )\\ Ax&= \Delta x\\ C(t)u(t)(\xi )&= \lambda (t)\beta (t,\xi )\\ \varphi (\theta )(\xi )&= \psi (\theta ,\xi ),\quad \theta \in [-r,0],\ \ \xi \in \Omega .\\ f(t,\varphi )(\xi )&= g(t,\varphi (-r)(\xi ))\quad {\text {for}}\ t\in [0,b]\ {\text {and}}\ \xi \in \Omega \\ (B(t)x)(\xi )&= h(t)\Delta x(t)(\xi )\quad {\text {for}}\ \ t\in [0,b],\ {\text {and}}\ \ \xi \in \Omega . \end{aligned}$$

Then, Eq. (10) can be transformed into the following abstract form:

11 $$\begin{aligned} \left\{ \begin{array}{l} x'(t)=Ax(t) + {\int _0^tB(t-s)x(s)ds} + f(t,x_t)+C(t)u(t)\quad {\text {for}}\ t\in I, \\ x_0=\varphi \in {\mathcal {C}}([-r,0],X), \end{array} \right. \end{aligned}$$

where *X* is a Banach space.

An example of a material with memory is *Shape-memory polymers* (SMPs), which are polymeric smart materials that have the ability to return from a deformed state (temporary shape) to their original (permanent) shape induced by an external stimulus (trigger), such as temperature change. That is they act adaptively to their environment, they can easily be shaped into different forms at a low temperature, but return to their original shape on heating.

In order to evaluate the performance of a system quantatively, the designer selects a performance measure or a cost function. In certain cases, the statement of the problem may clearly indicate what to select for a cost function, whereas in other cases, the selection of a cost function is a subjective matter. It will be assume that the cost function of a system is evaluated by a function of the form

$$J=\int _0^bL(\psi (t), x(t),u(t),t)dt,$$

where *L* is a scalar function.

Suppose the objective in this example is to make the material take a particular form, with minimum heating. The optimal control problem consists in solving the following: Find an admissible control $u^*$ which causes the system

$$x'(t)=Ax(t) + { \int _0^tB(t-s)x(s)ds} + f(t,x_t)+C(t)u(t)\quad {\text {for}}\ t\in I$$

to follow an admissible state $x^*$ that minimize the cost function

$${\mathcal {J}}(u):= {\int _0^b{\mathcal {L}}\left( t,\,x_t^u,\,x^u(t),\,u(t)\right) \,dt},$$

where $x^u$ denotes the mild solution of (11) corresponding to the control $u\in {\mathcal {U}}_{ad}$, the space of admissible controls. $u^*$ is called an optimal control and $x^*$ is called an optimal state.

Equation (11) has been studied by many authors [see e.g., Ezzinbi et al. (2009) and the references contained in it]. But to the best of our knowledge, this equation has never been considered for optimal control.

The rest of the paper is organized as follows: In second section, we present some basic definitions and preliminaries results, which will be used in the subsequent sections. In third section, we obtain an a priori estimation of mild solutions of Eq. (1). In fourth section, sufficient conditions are established for the existence and uniqueness of mild solutions of Eq. (1), by applying a well known fixed point theorem, and extension by continuity techniques. In fifth section, we investigate the existence of optimal controls of a Lagrange optimal control problem for Eq. (1). Finally, the last section, an example is given to illustrate the main results of this work.

## Resolvent operators and Balder’s theorem

In this section we introduce some definitions and Lemmas that will be used throughout the paper.

A measurable function $x:I\rightarrow X$ is Bochner integrable if and only if $\Vert x\Vert$ is Lebesgue integrable. We denote by $L^1(I,X)$ the Banach space of functions $x:I\rightarrow X$ which are Bochner integrable normed by

$$\begin{aligned} \Vert x\Vert =\int _0^b\Vert x(t)\Vert dt. \end{aligned}$$

Consider the following linear homogeneous equation:

12 $$\begin{aligned} \left\{ \begin{array}{l} x'(t)=Ax(t)+{ \int _0^tB(t-s)x(s)ds}\quad {\text {for}} \ \ t\ge 0 \\ x(0)=x_0\in X, \end{array} \right. \end{aligned}$$

where *A* and *B*(*t*) are closed linear operators on a Banach space *X*.

In the sequel, we assume *A* and $\left( B(t)\right) _{t\ge 0 }$ satisfy the following conditions:

### $(\mathbf{H_1})$

*A* is a densely defined closed linear operator in *X*. Hence ${\mathcal {D}}(A)$ is a Banach space equipped with the graph norm defined by, $|y|=\Vert Ay\Vert +\Vert y\Vert$ which will be denoted by $(X_1,|\cdot |)$.

### $(\mathbf{H_2})$

$\left( B(t)\right) _{t\ge 0 }$ is a family of linear operators on *X* such that *B*(*t*) is continuous when regarded as a linear map from $(X_1,|\cdot |)$ into $(X,\Vert \cdot \Vert )$ for almost all $t\ge 0$ and the map $t\mapsto B(t)y$ is measurable for all $y\in X_1$ and $t\ge 0$, and belongs to $W^{1,1}({\mathbb {R}}^{+},X)$. Moreover there is a locally integrable function $b:{\mathbb {R}}^{+}\rightarrow {\mathbb {R}}^{+}$ such that

$$\Vert B(t)y\Vert \ \le \ b(t)|y|\ \quad \mathrm{and}\ \quad \left\| \frac{d}{dt}B(t)y\right\| \ \le \ b(t)|y| .$$

### Remark 1

Note that $(\mathbf{H_2})$ is satisfied in the modelling of Heat Conduction in materials with memory and viscosity. More details can be found in Liang et al. (2008).

Let ${\mathcal {L}}(X)$ be the Banach space of bounded linear operators on *X*,

### **Definition 1**

(Ezzinbi et al. 2009) A resolvent operator $\left( R(t)\right) _{t\ge 0 }$ for Eq. (12) is a bounded operator valued function

$$R :\ [0,+\infty )\ \longrightarrow \ {\mathcal {L}}(X)$$

such that

(i) $R(0)=Id_X$ and $\Vert R(t)\Vert \le Ne^{\beta t}$ for some constants *N* and $\beta$.
(ii) For all $x\in X$, the map $t\mapsto R(t)x$ is continuous for $t\ge 0$.
(iii) Moreover for $x\in X_1$, $R(\cdot )x\,\in \,{\mathcal {C}}^1({\mathbb {R}}^{+};X)\cap {\mathcal {C}}({\mathbb {R}}^{+};X_1)$ and
  $$\begin{aligned} R'(t)x&= AR(t)x+\int _0^tB(t-s)R(s)xds\\&= R(t)Ax+\int _0^tR(t-s)B(s)xds. \end{aligned}$$

Observe that the map defined on ${\mathbb {R}}^{+}$ by $t\mapsto R(t)x_0$ solves Eq. (12) for $x_0\in {\mathcal {D}}(A)$.

### **Theorem 2**

(Grimmer 1983) *Assume that*$(H_1)$*and*$(H_2)$*hold. Then, the linear Eq.* (12) *has a unique resolvent operator*$\left( R(t)\right) _{t\ge 0 }$.

### Remark 2

In general, the resolvent operator $\left( R(t)\right) _{t\ge 0}$ for Eq. (12) does not satisfy the semigroup law, namely,

$$R(t+s) \ \ne \ R(t)R(s) \qquad {\text{ for }} {\text{ some}} \ \ t,\quad s >0 .$$

The following Theorem is needed in the proof of the existence of optimal controls.

### **Theorem 3**

(Balder 1987) *Let*$(\varSigma ,{\mathcal {F}},\mu )$*be a finite nonatomic measure space*, $(Y,\Vert \cdot \Vert )$*a separable Banach space*, *and*$(V,|\cdot |)$*a separable reflexive Banach space*, *and*$V'$*its dual. Let*$\theta : \varSigma \times Y\times V\rightarrow (-\infty ,+\infty ]$*be a given*${\mathcal {F}}\times {\mathcal {L}}(Y\times V)$- *measurable function*. *The associated integral functional*$I_{\theta }:L_Y^1\times L_V^1\rightarrow [-\infty ,+\infty ]$*is defined by:*

$$I_{\theta }(x,v)=\int _{\varSigma }\theta (t,x(t),v(t))\mu (dt),$$

*where*$L_Y^1$*denotes the space of all absolutely summable functions from*$\varSigma$*toY*.

*The following three conditions*

(i) $\theta (t,\cdot ,\cdot )$*is sequencially lower semicontinuous on*$X\times V,\ \mu$-*a.e.*,
(ii) $\theta (t,x,\cdot )$*is convex onVfor*$x\in Y$, $\mu$-*a.e.*,
(iii) *There exist*$\sigma >0$*and*$\psi \in L_{{\mathbb {R}}}^1$*such that*
  $$\begin{aligned} \theta (t,x,v)\ge \psi (t)-\sigma (\Vert x\Vert +|v|)\quad for\; all\ \ x\in Y,\quad v\in V,\quad \mu {\text {-a.e}}., \end{aligned}$$

*are sufficient for sequential strong–weak lower semicontinuity*$I_{\theta }$*on*$L_Y^1\times L_V^1$. *Moreover, they are also necessary, provided that*$I_{\theta }(\overline{x},\overline{v})<+\infty$*for some*$\overline{x}\in L_Y^1,\overline{v}\in L_V^1$.

### **Theorem 4**

(Mazur’s theorem) *LetZbe a Banach space andGbe a convex and closed set inZ*. *ThenGis weakly closed inZ*.

## Existence of mild solutions for Eq. (1)

We make the following assumptions.

### $\mathbf{(H_3)}$

 The function $f:\, I\times {\mathcal {C}}\rightarrow X$ satisfies the following conditions:

(i) $f(\cdot ,\psi )$ is measurable for $\psi \in {\mathcal {C}}$,
(ii) For any $\rho >0$, there exists $L_f(\rho )>0$ such that
  $$\begin{aligned} \Vert f(t,\psi _1)-f(t,\psi _2)\Vert \le L_f(\rho )\Vert \psi _1-\psi _2\Vert \quad {\text {for}}\ \Vert \psi _1\Vert \le \rho ,\ \Vert \psi _2\Vert \le \rho \ {\text {and}}\ t\in [0,b], \end{aligned}$$
(iii) There exists $a_f>0$ such that
  $$\begin{aligned} \Vert f(t,\psi )\Vert \le a_f(1+\Vert \psi \Vert )\quad {\text {for all}}\ \ \psi \in {\mathcal {C}}\ \ {\text {and}}\ \ \ \ t\in [0,b]. \end{aligned}$$

### $\mathbf{(H_4)}$

 Let *U* be the separable reflexive Banach space from which the control *u* takes values and assume $C\in L^{\infty }(I;{\mathcal {L}}(U,X))$.

### $\mathbf{(H_5)}$

The multivalued map $\varGamma :I\rightarrow 2^U{\setminus }\{\emptyset \}$ has closed, convex, and bounded values, $\varGamma$ is graph measurable, and $\varGamma (\cdot )\subseteq \Omega$ where $\Omega$ is a bounded set in *U*.

We denote by ${\mathcal {U}}_{ad}$ the set of admissible controls defined by:

$$\begin{aligned} {\mathcal {U}}_{ad}=\left\{ u:I\rightarrow U\,{\text {such that}}\,u\ {\text {is measurable and}}\ u(t)\in \varGamma (t),\quad a.e.\right\} . \end{aligned}$$

Then, we have the following:

### **Theorem 5**

Wang et al. (2012) ${\mathcal {U}}_{ad}\ne \emptyset$*and*${\mathcal {U}}_{ad}\subset L^2(I,U)$*is bounded, closed and convex. Also*, $Cu\in L^2(I,U)\ \ {\text {for all}}\ \ u\in {\mathcal {U}}_{ad}.$

### **Definition 6**

Let $u\in {\mathcal {U}}_{ad}$ and $\varphi \in {\mathcal {C}}$. A function $x\in {\mathcal {C}}([-r,b],X)$ is called a mild solution of Eq. (1) if

13 $$\begin{aligned} x(t)=\left\{ \begin{array}{l} R(t)\varphi (0)+ {\int _0^tR(t-s)\left[ f(s,x_s)+C(s)u(s)\right] \,ds}\quad {\text {for}}\ t\in I \\ \varphi (t)\quad {\text {for}}\ \ -r\le t\le 0. \end{array} \right. \end{aligned}$$

We have the following Theorem on existence of mild solutions to Eq. (1) with respect to a given control $u\in {\mathcal {U}}_{ad}$.

### **Theorem 7**

*Assume that* H_1_–H_5_*hold. Then for each*$u\in {\mathcal {U}}_{ad}$, *Eq.* (1) *has a unique mild solution on*$[-r,b]$.

### Proof

Let $b_1\le b,\ \rho >0$, and $\psi \in {\mathcal {C}}$ such that $\Vert \psi \Vert \le \rho$. For $t\in [0,b_1]$, we have by the local Lipschitz condition on *f* that

$$\begin{aligned} \Vert f(t,\psi )\Vert \le L_f(\rho )\Vert \psi \Vert +\Vert f(t,0)\Vert \le L_f(\rho )\rho +\sup _{s\in [0,b_1]}\Vert f(s,0)\Vert . \end{aligned}$$

$b_1$ will be chosen sufficiently small enough to get the local existence of mild solutions.

Let $$\varphi \in {\mathcal {C}},\ \rho =\Vert \varphi \Vert +1$$ and $$\rho ^*=L_f(\rho )\rho +\sup _{s\in [0,b_1]}\Vert f(s,0)\Vert$$.

We define the following space

$$\begin{aligned} E_{\varphi }=\left\{ x\in {\mathcal {C}}([-r,b_1];X)\ {\text {such that}} \ x(\theta )=\varphi (\theta )\quad {\text {for}}\ \theta \in [-r,0]\ {\text {and}}\ \sup _{s\in [0,b_1]}\Vert x(s)-\varphi (0)\Vert \le 1\right\} . \end{aligned}$$

For $x\in E_{\varphi }$, one can see that $\Vert x_t\Vert \le 1+\Vert \varphi \Vert =\rho$.

Then, $E_{\varphi }$ is a closed subset of ${\mathcal {C}}([-r,b_1];X)$ which is endowed with the uniform norm topology.

Let

$$\begin{aligned} M_b= \sup _{t\in [0,b]}\Vert R(t)\Vert . \end{aligned}$$

Define the operator $K:\,E_{\varphi }\rightarrow {\mathcal {C}}([-r,b_1];X)$ by

$$\begin{aligned} (Kx)(t)=\left\{ \begin{array}{l} R(t)\varphi (0)+ {\int _0^tR(t-s)\left[ f(s,x_s)+C(s)u(s)\right] \,ds}\quad {\text {for}}\ t\in [0,b_1] \\ \varphi (t) \quad {\text {for}}\ \ t\in [-r,0] \end{array} \right. \end{aligned}$$

We claim that $K\left( E_{\varphi }\right) \subset E_{\varphi }$. In fact let $x\in E_{\varphi }$ and $t\in [0,b_1]$. Then,

$$\begin{aligned} \Vert (Kx)(t)-\varphi (0)\Vert& \le \Vert R(t)\varphi (0)-\varphi (0)\Vert \\&\quad + {\int _0^t\left\| R(t-s)\left[ f(s,x_s)+C(s)u(s)\right] \right\| \,ds}\\ &\le \Vert R(t)\varphi (0)-\varphi (0)\Vert +M_b\,\rho ^*t+M_b\,\Vert C\Vert \,\Vert u\Vert _{L^2}\sqrt{t}. \end{aligned}$$

Now, choose $b_1$ sufficiently small such that

14 $$\begin{aligned} \sup _{s\in [0,b_1]}\left\{ \Vert R(s)\varphi (0)-\varphi (0)\Vert +M_b\,\rho ^*s+M_b\,\Vert C\Vert \,\Vert u\Vert _{L^2}\sqrt{s}\right\} <1. \end{aligned}$$

Consequently,

$$\begin{aligned} \Vert (Kx)(t)-\varphi (0)\Vert \le \Vert R(t)\varphi (0)-\varphi (0)\Vert +M_b\,\rho ^*t+M_b\,\Vert C\Vert \,\Vert u\Vert _{L^2}\sqrt{t}<1\ \ {\text {for}}\ t\in [0,b_1]. \end{aligned}$$

Hence, $$K\left( E_{\varphi }\right) \subset E_{\varphi }$$.

Let $x,\, y\in E_{\varphi }$ and $t\in [0,b_1]$. Then, $\Vert x_s\Vert ,\ \Vert y_s\Vert \le \rho$ for $s\in [0,b_1]$ and we have

$$\begin{aligned} \Vert (Kx)(t)-(Ky)(t)\Vert & \le M_b\int _0^t\Vert f(s,x_s)-f(s,y_s)\Vert \,ds\\ & \le M_b\,L_f(\rho )\int _0^t\Vert x_s-y_s\Vert \,ds\\ & \le M_b\,L_f(\rho )\int _0^t\sup _{\tau \in [0,s]}\Vert x(\tau )-y(\tau )\Vert \,d\tau \\& \le M_b\,L_f(\rho )\,b_1\,\Vert x-y\Vert \end{aligned}$$

Now, since

$$\begin{aligned} M_b\,L_f(\rho )\,b_1\le M_b\,\rho ^*\,b_1<\sup _{s\in [0,b_1]}\left\{ \Vert R(s)\varphi (0)-\varphi (0)\Vert +M_b\,\rho ^*s+M_b\,\Vert C\Vert \,\Vert u\Vert _{L^2}\sqrt{s}\right\} . \end{aligned}$$

Condition (14) implies that

$$\begin{aligned} M_b\,L_f(\rho )\,b_1<1. \end{aligned}$$

Thus, *K* is a strict contraction on $E_{\varphi }$. It follows from the contraction mapping principle that *K* has a unique fixed point $x\in E_{\varphi }$, which is the unique mild solution of Eq. (1) with respect to *u* on $[-r,b_1]$. $\square$

Using the same arguments, we can show that *x* can be extended to a maximal interval of existence $[0,t_{\max }[$.

### **Lemma 8**

(Ezzinbi et al. 2009) *If*$t_{\max }<b$, *then*, $\limsup _{t\rightarrow t_{\max }}\Vert x(t)\Vert =\infty$.

We show that $t_{\max }=b$. Assume on the contrary that $t_{\max }<b$. Then for $t\in [0,t_{\max }]$ we have that

$$\begin{aligned} x(t)=R(t)\varphi (0)+ {\int _0^tR(t-s)\left[ f(s,x_s)+C(s)u(s)\right] \,ds}. \end{aligned}$$

It follows that

$$\begin{aligned} \Vert x(t)\Vert & \le M_b\Vert \varphi (0)\Vert +M_b\int _0^t\Vert f(s,x_s)\Vert \,ds+M_b\int _0^t\Vert C(s)u(s)\Vert \,ds\\ & \le M_b\Vert \varphi \Vert +M_b\,t_{\max }\,a_f+M_ba_f\int _0^t\Vert x_s\Vert \,ds+M_b\Vert C\Vert \int _0^t\Vert u(s)\Vert \,ds\\ & \le M_b\Vert \varphi \Vert +M_b\,t_{\max }\,a_f+M_b\,\sqrt{t_{\max }}\,\Vert C\Vert \Vert u\Vert _{L^2}+M_b\,a_f\int _0^t\Vert x_s\Vert \,ds\\ & \le M_b\Vert \varphi \Vert +M_b\,t_{\max }\,a_f+M_b\,\sqrt{t_{\max }}\,\Vert C\Vert \Vert u\Vert _{L^2}+M_b\,a_f\int _0^t\sup _{\tau \in [-r,s]}\Vert x(\tau )\Vert \,d\tau \end{aligned}$$

This implies that

$$\begin{aligned} \sup _{s\in [-r,t]}\Vert x(s)\Vert \le M_b\Vert \varphi \Vert +M_b\,t_{\max }\,a_f+M_b\,\sqrt{t_{\max }}\,\Vert C\Vert \Vert u\Vert _{L^2}+M_b\,a_f\int _0^t\sup _{\tau \in [-r,s]}\Vert x(\tau )\Vert \,d\tau \end{aligned}$$

It follows by Gronwall’s inequality that

$$\begin{aligned} \Vert x(t)\Vert \le \beta ^*e^{M_b\,a_f\,t}\quad {\text {for}}\ t\in [0,t_{\max }], \end{aligned}$$

where $$\beta ^*=M_b\Vert \varphi \Vert +M_b\,t_{\max }\,a_f+M_b\,\sqrt{t_{\max }}\,\Vert C\Vert \Vert u\Vert _{L^2}$$.

Thus

$$\begin{aligned} \lim _{t\rightarrow t_{\max }}\Vert x(t)\Vert \le \beta ^*e^{M_b\,a_f\,t_{\max }}<\infty . \end{aligned}$$

This contradicts Lemma 8. Therefore, $t_{\max }=b$ and hence, Eq. (1) has a unique mild solution on $[-r,b]$.

## Continuous dependence and existence of the optimal control solving Eq. (1)

In this section, we discuss the continuous dependence of the mild solutions of Eq. (1) on the controls and initial states, and the existence of solutions of the Lagrange problem associated to Eq. (1).

We have the following a priori estimation.

### **Lemma 9**

*Suppose (H*_*1*_*)–(H*_*3*_*) holds and assume that Eq.* (1) *has a mild solution*$x_u$*on*$[-r,b]$*with respect to*$u\in {\mathcal {U}}_{ad}$. *Then, there exists a constant*$\rho >0$*independent ofusuch that*$\Vert x_u(t)\Vert \le \rho \quad {\text {for}}\ t\in [0,b]$, ($\rho$*depends only on*${\mathcal {U}}_{ad}$*and*$\varphi$).

### Proof

Let $\varphi \in {\mathcal {C}}$. We define the following space

$$\begin{aligned} E_b=\left\{ x:[0,b]\rightarrow X\ {\text {continuous such that}}\ x(0)=\varphi (0)\right\} . \end{aligned}$$

For $x\in E_b$, we define its continuous extension $\widetilde{x}$ over $[-r,b]$ by

$$\begin{aligned} \widetilde{x}(t)=\left\{ \begin{array}{l} x(t)\quad {\text {for}}\ \ t\in [0,b] \\ \varphi (t) \quad {\text {for}}\ \ t\in [-r,0] \end{array} \right. \end{aligned}$$

For $\varphi \in {\mathcal {C}}$, we define the function $y\in {\mathcal {C}}([0,b],X)$ by $y(t)= R(t)\varphi (0)$ and its extension $\widetilde{y}$ in ${\mathcal {C}}([-r,b],X)$ by

$$\begin{aligned} \widetilde{y}(t)=\left\{ \begin{array}{ll} y(t)&\quad {\text {for}}\ \ t\in [0,b] \\ \varphi (t)&\quad {\text {for}}\ \ t\in [-r,0]\\ \end{array} \right. \end{aligned}$$

For each $z\in {\mathcal {C}}([0,b],X)$, set $\widetilde{x}(t)=\widetilde{z}(t)+\widetilde{y}(t)$, where $\widetilde{z}$ is the extension by zero of the function *z* on $[-r,0]$. Observe that *x* satifies (13) if and only if $z(0)=0$ and

$$\begin{aligned} z(t)= \int _0^tR(t-s)\left[ f(s,\widetilde{z}_s+\widetilde{y}_s)+C(s)u(s)\right] \,ds\quad {\text {for}}\ t\in [0,b], \end{aligned}$$

Let

$$\begin{aligned} M_b= \sup _{t\in [0,b]}\Vert R(t)\Vert \quad {\text {and}}\quad \Vert C\Vert =\sup _{t\in I}\Vert C(t)\Vert _{{\mathcal {L}}(U,X)}. \end{aligned}$$

Since ${\mathcal {U}}_{ad}$ is bounded, let $\widetilde{K}>0$ be such that $\Vert u\Vert _{L^2}\le \widetilde{K}$ for all $u\in {\mathcal {U}}_{ad}$.

$$\begin{aligned} \Vert z(t)\Vert & \le M_b\int _0^t\Vert f(s,\widetilde{z}_s+\widetilde{y}_s)\Vert \,ds+M_b\int _0^t\Vert C(s)u(s)\Vert \,ds\\ & \le M_b\,b\,a_f+M_ba_f\int _0^t\Vert \widetilde{z}_s+\widetilde{y}_s\Vert \,ds+M_b\Vert C\Vert \int _0^b\Vert u(s)\Vert \,ds\\& \le M_b\,b\,a_f+M_b\,\sqrt{b}\,\Vert C\Vert \Vert u\Vert _{L^2}+M_b\,a_f\int _0^t\Vert \widetilde{z}_s+\widetilde{y}_s\Vert \,ds\\ & \le M_b\,b\,a_f+M_b\,\sqrt{b}\,\Vert C\Vert \widetilde{K}+M_b\,a_f\int _0^t\Vert \widetilde{z}_s+\widetilde{y}_s\Vert \,ds, \end{aligned}$$

Thus

15 $$\begin{aligned} \Vert z(t)\Vert & \le M_b\,b\,a_f+M_b\,\sqrt{b}\,\Vert C\Vert \widetilde{K}+M_b\,a_f\int _0^t\Vert \widetilde{z}_s+\widetilde{y}_s\Vert \,ds. \\ \Vert \widetilde{z}_s+\widetilde{y}_s\Vert & \le \Vert \widetilde{z}_s\Vert +\Vert \widetilde{y}_s\Vert \\ & \le \Vert \widetilde{z}_s\Vert +M_b\Vert \varphi \Vert \\ & \le \sup _{\tau \in [0,s]}\Vert z(\tau )\Vert +M_b\Vert \varphi \Vert \end{aligned}$$

This implies that (15) can be rewritten as follows

$$\begin{aligned} \sup _{s\in [0,t]}\Vert z(s)\Vert & \le M_b\,b\,a_f+M_b\,\sqrt{b}\,\Vert C\Vert \widetilde{K}+M_b^2\,a_f\,b\,\Vert \varphi \Vert +M_b\,a_f\int _0^t\sup _{\tau \in [0,s]}\Vert z(\tau )\Vert \,d\tau \\&= M+M_b\,a_f\int _0^t\sup _{\tau \in [0,s]}\Vert z(\tau )\Vert \,d\tau . \end{aligned}$$

with $$M=M_b\,b\,a_f+M_b\,\sqrt{b}\,\Vert C\Vert \widetilde{K}+M_b^2\,a_f\,b\,\Vert \varphi \Vert$$.

It follows by Gronwall’s inequality that

$$\begin{aligned} \Vert z(t)\Vert \le Me^{b\,a_f\,M_b}=:\widetilde{M}. \end{aligned}$$

As a result, for $t\in I$, we have

$$\begin{aligned} \Vert x_u(t)\Vert & \le \Vert z(t)\Vert +\Vert R(t)\varphi (0)\Vert \\ & \le \widetilde{M}+M_b\Vert \varphi \Vert :=\rho \end{aligned}$$

That is $\Vert x_u(t)\Vert \le \rho \quad {\text {for all}}\ t\in I$. This completes the proof of the Lemma. $\square$

We have the following theorem on continuous dependence of the mild solutions of Eq. (1) on the controls and initial states.

### **Theorem 10**

*For all*$\lambda >0$, *there exists*$\gamma ^*(\lambda )>0$*such that for all*$\varphi ^1,\ \varphi ^2\in B(0,\lambda )$,

$$\begin{aligned} \Vert x^1_t-x^2_t\Vert \le \gamma ^{*}\left( \Vert \varphi ^1-\varphi ^2\Vert +\Vert u^1-u^2\Vert _{L^2}\right) \quad { {for}}\ \ t\in [0,b], \end{aligned}$$

*where*

16 $$\begin{aligned} x^i(t)=\left\{ \begin{array}{l} R(t)\varphi ^i(0)+ {\int _0^tR(t-s)\left[ f(s,x^i_s)+C(s)u^i(s)\right] \,ds}\quad {{for}}\ t\in I \\ \varphi ^i(t)\quad { {for}}\ \ -r\le t\le 0, \end{array} \right. \end{aligned}$$

*and*$$u^i\in {\mathcal {U}}_{ad}$$, for *i* = 1, 2.

### Proof

Let $x^i$, for *i* = 1, 2, be two mild solutions of Eq. (1), corresponding to the controls $u^i\in {\mathcal {U}}_{ad}$ and $\lambda >0$ such that $\varphi ^1,\ \varphi ^2\in B(0,\lambda )$.

$$\begin{aligned} x^i(t)=\left\{ \begin{array}{l} R(t)\varphi ^i(0)+ {\int _0^tR(t-s)\left[ f(s,x^i_s)+C(s)u^i(s)\right] \,ds}\quad {{for}}\ t\in I \\ \varphi ^i(t)\quad {{for}}\ \ -r\le t\le 0, \end{array} \right. \end{aligned}$$

From the proof of Lemma 9, one can see that for $\rho _{\lambda }=\widetilde{M}+M_b\lambda >0$, we have $\Vert x^i_s\Vert \le \rho _{\lambda },\ i=1,\,2.$

Now, for $t\in [0,b]$, we have

$$\begin{aligned} \Vert x^1(t)-x^2(t)\Vert & \le M_b\Vert \varphi ^1(0)-\varphi ^2(0)\Vert +M_b\int _0^t\Vert f(s,x^1_s)-f(s,x^2_s)\Vert \,ds\\&\quad + M_b\int _0^t\Vert C(s)u^1(s)-C(s)u^2(s)\Vert \,ds\\ & \le M_b\Vert \varphi ^1-\varphi ^2\Vert +M_bL_f(\rho _{\lambda })\int _0^t\left( \Vert x^1_s-x^2_s\Vert \right) \,ds\\&\quad + M_b\int _0^t\Vert C(s)u^1(s)-C(s)u^2(s)\Vert \,ds\\& \le M_b\Vert \varphi ^1-\varphi ^2\Vert +M_bL_f(\rho _{\lambda })\int _0^t\Vert x^1_s-x^2_s\Vert \,ds\\&\quad + M_bL_f(\rho _{\lambda })\,\sqrt{b}\,\Vert C\Vert \,\Vert u^1-u^2\Vert _{L^2} \end{aligned}$$

That is

17 $$\begin{aligned} \Vert x^1(t)-x^2(t)\Vert& \le M_b\Vert \varphi ^1-\varphi ^2\Vert +M_bL_f(\rho _{\lambda })\int _0^t\Vert x^1_s-x^2_s\Vert \,ds\\&\quad + M_bL_f(\rho _{\lambda })\,\sqrt{b}\,\Vert C\Vert \,\Vert u^1-u^2\Vert _{L^2} \end{aligned}$$

But we have that

$$\begin{aligned} \Vert x^1_s-x^2_s\Vert \le \sup _{\tau \in [-r,s]}\Vert x^1(\tau )-x^2(\tau )\Vert . \end{aligned}$$

It follows that

$$\begin{aligned} \sup _{s\in [-r,t]}\Vert x^1(s)-x^2(s)\Vert& \le M_b\Vert \varphi ^1-\varphi ^2\Vert +M_bL_f(\rho )\,\sqrt{b}\,\Vert C\Vert \,\Vert u^1-u^2\Vert _{L^2}\\&\quad + M_bL_f(\rho _{\lambda })\int _0^t \sup _{\tau \in [-r,s]}\Vert x^1(\tau )-x^2(\tau )\Vert \,d\tau . \end{aligned}$$

By Gronwall’s inequality, we have that

$$\begin{aligned} \sup _{s\in [-r,t]}\Vert x^1(s)-x^2(s)\Vert\le & {} \left[ M_b\Vert \varphi ^1-\varphi ^2\Vert +M_bL_f(\rho _{\lambda })\,\sqrt{b}\,\Vert C\Vert \,\Vert u^1-u^2\Vert _{L^2}\right] e^{M_bL_f(\rho _{\lambda })b}. \end{aligned}$$

This implies that

$$\begin{aligned} \Vert x^1_t-x^2_t\Vert\le & {} \left[ M_b\Vert \varphi ^1-\varphi ^2\Vert +M_bL_f(\rho _{\lambda })\,\sqrt{b}\,\Vert C\Vert \,\Vert u^1-u^2\Vert _{L^2}\right] e^{M_bL_f(\rho _{\lambda })b}, \end{aligned}$$

Let

$$\begin{aligned} \gamma ^*(\lambda ):=\max \left\{ M_b\,e^{M_bL_f(\rho _{\lambda })b},\ M_bL_f(\rho _{\lambda })\,\sqrt{b}\,\Vert C\Vert \,e^{M_bL_f(\rho _{\lambda })b}\right\} . \end{aligned}$$

Then, we have that

$$\begin{aligned} \Vert x^1_t-x^2_t\Vert \le \gamma ^*(\lambda )\left( \Vert \varphi ^1-\varphi ^2\Vert +\Vert u^1-u^2\Vert _{L^2}\right) \quad {\text {for}}\ t\in [0,b]. \end{aligned}$$

And the proof is complete. $\square$

Now, we study the existence of solutions to the following Lagrange problem

$$\begin{aligned} ({\mathcal {LP}})\left\{ \begin{array}{l} {\text {Find a control}}\ u^0\in {\mathcal {U}}_{ad}\ \ {\text {such that}}\\ {\mathcal {J}}(u^0)\le {\mathcal {J}}(u)\quad {\text {for all}}\ \ u\in {\mathcal {U}}_{ad}, \end{array} \right. \end{aligned}$$

where

$$\begin{aligned} {\mathcal {J}}(u):= {\int _0^b{\mathcal {L}}\left( t,\,x_t^u,\,x^u(t),\,u(t)\right) \,dt}, \end{aligned}$$

and $x^u$ denotes the mild solution of (1) corresponding to the control $u\in {\mathcal {U}}_{ad}$ and the initial data $\varphi$.

For the existence of solutions to problem $({\mathcal {LP}})$, we make the following assumptions.$\mathbf{(H_L)}$

(i) The functional ${\mathcal {L}}\,:\,I\times {\mathcal {C}}\times X\times U\rightarrow \mathbb {R}\cup \{\infty \}$ is Borel measurable.
(ii) ${\mathcal {L}}(t,\,\cdot ,\,\cdot ,\,\cdot )$ is sequencially lower semicontinuous on ${\mathcal {C}}\times X\times U$ for almost all $t\in I$.
(iii) ${\mathcal {L}}(t,\,\psi ,\,y,\,\cdot )$ is convex on *U* for each $\psi \in {\mathcal {C}},\ \ y\in X$ and almost all $t\in I$.
(iv) There exist constants $\nu ,\ \beta \ge 0,\ \gamma >0$, and $\mu \in L^1(I)$ nonnegative such that
  $$\begin{aligned} {\mathcal {L}}(t,\,\psi ,\,y,\,u)\ge \mu (t)+\nu \Vert \psi \Vert +\beta \Vert y\Vert +\gamma \Vert u\Vert . \end{aligned}$$

We have the following result on the existence of optimal controls for problem $({\mathcal {LP}})$.

### **Theorem 11**

*Assume that hypotheses (H*_*1*_*)–(H*_*5*_*)and (H*_*L*_*) hold.Then the Lagrange problem*$({\mathcal {LP}})$*admits at least one optimal pair, that is there exists an admissible control pair*$(x^0,u^0)\in {\mathcal {C}}([-r,b],X)\times {\mathcal {U}}_{ad}$*such that*

$$\begin{aligned} {\mathcal {J}}(u^0)&= {\int _0^b{\mathcal {L}}\left( t,\,x_t^0,\,x^0(t),\,u^0(t)\right) \,dt}\\& \le {\int _0^b{\mathcal {L}}\left( t,\,x_t^u,\,x^u(t),\,u(t)\right) \,dt}\\& ={\mathcal {J}}(u)\ \ {\text {for}}\ u\in {\mathcal {U}}_{ad}. \end{aligned}$$

### Proof

If $\inf \left\{ {\mathcal {J}}(u)\,:\,u\in {\mathcal {U}}_{ad}\right\} =\infty$, we are done. $\square$

Without loss of generality, assume that $\inf \left\{ {\mathcal {J}}(u)\,:\,u\in {\mathcal {U}}_{ad}\right\} =\delta <\infty$.

Suppose that $\delta =-\infty$, then for each $n\in {\mathbb {N}}$, there exists $(u^n)_{n\ge 1}\subset {\mathcal {U}}_{ad}$ such that

$${\mathcal {J}}(u^n)<-n \quad \quad \hfill(*)$$

Boundedness of ${\mathcal {U}}_{ad}$ implies that $(u^n)_{n\ge 1}$ is bounded and so there exists a subsequence $(u^{n_k})_{k\ge 1}$ of $(u^n)_{n\ge 1}$ that converges weakly to some $u^0$ in $L^2(I,U)$, since $L^2(I,U)$ is reflexive. But ${\mathcal {U}}_{ad}$ is closed and convex, so by Mazur’s Theorem, it is weakly closed and therefore, $u^0\in {\mathcal {U}}_{ad}$. By hypothesis (H_L_), ${\mathcal {L}}(t,\,\psi ,\,y,\,\cdot )$ is weakly lower semicontinuous, so we have that

$$\begin{aligned} {\mathcal {L}}(t,\,\psi ,\,y,\,u^0)\le \liminf _{k\rightarrow \infty }{\mathcal {L}}(t,\,\psi ,\,y,\,u^{n_k})<-\infty , \end{aligned}$$

which implies that ${\mathcal {J}}(u^0)<-\infty$ using $(*)$. And this is a contradiction since ${\mathcal {J}}(u^0)\in \mathbb {R}\cup \{\infty \}$. Hence $\delta \in \mathbb {R}$.

Now by the definition of $\delta$, there exists a minimizing sequence, a feasible pair $((x^n,u^n))_{n\ge 1}\subset {\mathcal {S}}_{ad}$ such that

$$\begin{aligned} {\int _0^b{\mathcal {L}}\left( t,\,x_t^n,\,x^n(t),\,u^n(t)\right) \,dt}\longrightarrow \delta \quad {\text {as}}\ n\rightarrow \infty , \end{aligned}$$

where

$$\begin{aligned} {\mathcal {S}}_{ad}:=\left\{ (x,u)\,:\,x\ {\text {is a mild solution of equation}}~(1) {\text { corresponding to the control}}\ u\in {\mathcal {U}}_{ad}\right\} . \end{aligned}$$

Boundedness of ${\mathcal {U}}_{ad}$ and the fact that $L^2(I,U)$ is reflexive imply that $(u^n)_{n\ge 1}$ has a subsequence denoted for simplicity by $(u^{k})_{k\ge 1}$, that converges weakly to some $u^0$ in $L^2(I,U)$. But ${\mathcal {U}}_{ad}$ is closed and convex, so by Mazur’s Theorem, it is weakly closed and therefore, $u^0\in {\mathcal {U}}_{ad}$.

Let

$$\begin{aligned} x^k(t)=\left\{ \begin{array}{l} R(t)\varphi (0)+ {\int _0^tR(t-s)\left[ f(s,x^k_s)+C(s)u^k(s)\right] \,ds}\quad {\text {for}}\ t\in I \\ \varphi (t)\quad {\text {for}}\ \ -r\le t\le 0. \end{array} \right. \end{aligned}$$

denote the subsequence of $(x^n)_{n\ge 1}$ corresponding to the control sequence $(u^k)_{k\ge 1}$ and $x^0$ be the mild solution corresponding to the control $u^0\in {\mathcal {U}}_{ad}$. We show that $x^k\rightarrow x^0$.

For $t\in [0,b]$, we have

$$\begin{aligned} \left\| x^k(t)-x^0(t)\right\|& \le \int _0^t\left\| R(t-s)\left[ f(s,x_s^k)-f(s,x_s^0)\right] \right\| \,ds\\&\quad + \int _0^t\left\| R(t-s)\left[ C(s)u^k(s)-C(s)u^0(s)\right] \right\| \,ds\\ & \le M_bL_f(\rho )\int _0^t\left\| x_s^k-x_s^0\right\| \,ds+M_b\\&\quad \int _0^t\left\| C(s)u^k(s)-C(s)u^0(s)\right\| \,ds\\ & \le M_bL_f(\rho )\int _0^t\left\| x_s^k-x_s^0\right\| \,ds+M_b\,\sqrt{b}\\&\quad \left( \int _0^t\left\| C(s)u^k(s)-C(s)u^0(s)\right\| ^2\,ds\right) ^{\frac{1}{2}}\\ & \le M_bL_f(\rho )\int _0^t\left\| x_s^k-x_s^0\right\| \,ds+M_b\,\sqrt{b}\,\left\| Cu^k-Cu^0\right\| _{L^2(I,U)}\\ \end{aligned}$$

That is

18 $$\begin{aligned} \left\| x^k(t)-x^0(t)\right\| \le M_bL_f(\rho )\int _0^t\left\| x_s^k-x_s^0\right\| \,ds+M_b\,\sqrt{b}\,\left\| Cu^k-Cu^0\right\| _{L^2(I,U)} \end{aligned}$$

But we have that

$$\begin{aligned} \Vert x_s^k-x_s^0\Vert \le \sup _{\tau \in [0,s]}\Vert x^k(\tau )-x^0(\tau )\Vert . \end{aligned}$$

This implies that

$$\begin{aligned} \sup _{s\in [0,t]}\left\| x^k(s)-x^0(s)\right\| & \le M_b\,\sqrt{b}\,\left\| Cu^k-Cu^0\right\| _{L^2(I,U)}\\&\quad +M_bL_f(\rho )\int _0^t\sup _{\tau \in [0,s]}\left\| x^k(\tau )-x^0(\tau )\right\| \,d\tau . \end{aligned}$$

It follows from Gronwall’s inequality that

19 $$\begin{aligned} \left\| x^k(t)-x^0(t)\right\| \le M^{**}\left\| Cu^k-Cu^0\right\| _{L^2(I,U)},\ \ {\text {where}}\ \ M^{**}=M_b\,\sqrt{b}\,e^{M_b\,bL_f(\rho )}. \end{aligned}$$

We have the following Lemma.

### **Lemma 12**

(Wang et al. 2012) *Let*$(u^n)_{n\ge 1}\subset {\mathcal {U}}_{ad}$*and*$u^0\in {\mathcal {U}}_{ad}$*such that*$(u^n)_{n\ge 1}$*converges weakly to*$u^0$. *Then,*

$$\begin{aligned} \left\| Cu^k-Cu^0\right\| _{L^2(I,U)}\longrightarrow 0\ \ {\text {as}}\ k\rightarrow \infty ,\quad {\text {if}}\ \ C\in L^{\infty }(I;{\mathcal {L}}(U,X)). \end{aligned}$$

We have by (19) that

$$\begin{aligned} \left\| x^k-x^0\right\| \le M^{**}\left\| Cu^k-Cu^0\right\| _{L^2(I,U)}, \end{aligned}$$

and therefore, it follows by Lemma 12 that

$$\begin{aligned} x^k\longrightarrow x^0\ \ {\text {as}}\ k\rightarrow \infty . \end{aligned}$$

We note that (H_L_) implies the assumptions of Balder’s Theorem. Hence by using Balder’s Theorem, we can conclude that $(x_t,\,x,\,u)\mapsto {\int _0^b{\mathcal {L}}\left( t,\,x_t,\,x(t),\,u(t)\right) \,dt}$ is sequencially lower semicontinuous in the strong topology of ${\mathcal {C}}([-r,0],X)\times L^1(I,X)\times L^1(I,U)$.

Now, since ${\mathcal {C}}([-r,0],X)\times L^2(I,X)\times L^2(I,U)\subset {\mathcal {C}}([-r,0],X)\times L^1(I,X)\times L^1(I,U)$, ${\mathcal {J}}$ is also sequencially lower semicontinuous on ${\mathcal {C}}([-r,0],X)\times L^2(I,X)\times L^2(I,U)$, and in the strong topology of $L^1(I,E_{\varphi }\times X\times U)$.

Hence, ${\mathcal {J}}$ is weakly lower semicontinuous on $L^2(I,U)$, and since by (H_L_)-(iv), ${\mathcal {J}}>-\infty$, ${\mathcal {J}}$ attains its infimum at $u^0\in {\mathcal {U}}_{ad}$, that is

$$\begin{aligned} \delta =\lim _{k\rightarrow \infty } {\int _0^b{\mathcal {L}}\left( t,\,x_t^k,\,x^k(t),\,u^k(t)\right) \,dt}\ge {\int _0^b{\mathcal {L}}\left( t,\,x_t^0,\,x^0(t),\,u^0(t)\right) \,dt}={\mathcal {J}}(u^0)\ge \delta . \end{aligned}$$

Thus, $\delta ={\mathcal {J}}(u^0)$, and hence there exists an admissible control $u^0\in {\mathcal {U}}_{ad}$ such that

$${\mathcal {J}}(u^0)\le {\mathcal {J}}(u)\ \ {\text {for all}}\ \ u\in {\mathcal {U}}_{ad}.$$

This completes the proof.

We now illustrate our main result by the following example. We observe that in Wang et al. (2012), the Langrangian function ${\mathcal {L}}$ defined by the authors in the example does not satisfy condition (H_L_)–(iv), as they claimed. We correct that here.

## Example

Let $\Omega$ be bounded domain in ${\mathbb {R}}^n$ with smooth boundary and consider the following nonlinear integrodifferential equation.

20 $$\begin{aligned} \left\{ \begin{array}{ll} \frac{\partial v(t,\xi )}{\partial t} = \Delta v(t,\xi )+{ \int _0^t \zeta (t-s)\Delta v(s,\xi )\,ds}+\alpha (t)\sin (v^2(t-r,\xi ))+\beta (t) \omega (t,\xi )\\ \qquad \quad {\text {for}}\ t\in I=[0,1]\ {\text {and}}\ \xi \in \Omega \\ v(t,\xi )= 0\quad {\text {for}}\ t\in [0,1]\ {\text {and}}\ \xi \in \partial \Omega \\ v(\theta ,\xi ) = \phi (\theta ,\xi )\quad {\text {for}}\ \theta \in [-r,0]\ {\text {and}}\ \xi \in \Omega ,\ \end{array} \right. \end{aligned}$$

where $\alpha ,\ \beta \in {\mathcal {C}}([0,1];{\mathbb {R}})$, $\omega :I\times \Omega \rightarrow {\mathbb {R}}$ continuous in *t* and $\zeta \in W^{1,1}({\mathbb {R}}^{+},\mathbb {R}^{+})$ Let $X=U=L^2(\Omega )$.

For $\eta >0$, we define the set of admissible controls ${\mathcal {U}}_{ad}$ by

$${\mathcal {U}}_{ad}:=\left\{ u:I\rightarrow U\ {\text {such that}}\ u\ {\text {is measurable and}}\,\Vert u\Vert _{L^2(I,U)}\le \eta \right\},$$

where

$$\Vert u\Vert ^2_{L^2(I,U)}=\int _0^1\left( \int _{\Omega }u^2(s)(\xi )\,d\xi \right) ds.$$

We define $A:{\mathcal {D}}(A)\subset X\rightarrow X$ by:

$$\begin{aligned} \left\{ \begin{array}{l} {\mathcal {D}}(A)=H^2(\Omega )\cap H_{0}^1(\Omega )\\ Av\,=\,\Delta v\quad {\text {for}}\ v\in {\mathcal {D}}(A). \end{array} \right. \end{aligned}$$

### **Theorem 13**

[Theorem 4.1.2, p. 79 of Vrabie (2003)] *The linear operatorAdefined above, is the infinitesimal generator of aC*_0_-*semigroup on*$L^2(\Omega )$.

*A* generates a *C*_0_-semigroup $\left( T(t)\right) _{t\ge 0}$ on $L^2(\Omega )$.

Define

$$\begin{aligned} x(t)(\xi )\ =\ v(t,\xi ),\ \ \ x'(t)(\xi )\ =\ \frac{\partial v(t,\xi )}{\partial t},\ \ \ \omega (t,\xi )=u(t)(\xi ).\\ \varphi (\theta )(\xi )=\phi (\theta ,\xi )\quad {\text {for}}\ \theta \in [-r,0]\ {\text {and}}\ \xi \in \Omega .\\ f(t,\varphi )(\xi )\ =\ \alpha (t)\sin \left( (\varphi ^2(-r)(\xi ))\right) \quad {\text {for}}\ t\in [0,1]\ {\text {and}}\ \xi \in \Omega . \end{aligned}$$

$C(t):X\rightarrow X$ be defined by $\left( C(t)u(t)\right) (\xi )\,=\,C(t)u(t)(\xi )=\,\beta (t)\omega (t,\xi ).$

$$\begin{aligned} (B(t)x)(\xi )\ =\ \zeta (t)\Delta v(t,\xi )\quad {\text {for}}\ \ t\in [0,1],\ \ x\in {\mathcal {D}}(A)\ {\text {and}}\ \ \xi \in \Omega . \end{aligned}$$

Equation (20) is then transformed into the following form

21 $$\begin{aligned} \left\{ \begin{array}{l} x'(t)=Ax(t) + { \int _0^tB(t-s)x(s)ds} + f(t,x_t)+C(t)u(t)\ \ {\text {for}}\ t\in I=[0,1], \\ x_0=\varphi . \end{array} \right. \end{aligned}$$

One can see that, *f* satisfies (H_3_). Now we consider the following cost function:

$${\mathcal {J}}(u):= {\int _0^1{\mathcal {L}}\left( t,\,x_t^u,\,x^u(t),\,u(t)\right) dt},$$

where ${\mathcal {L}}\,:\,[0,1]\times {\mathcal {C}}([-r,0],L^2(\Omega ))\times L^2(\Omega )\times L^2(\Omega )\longrightarrow {\mathbb {R}}$ is defined by:

$${\mathcal {L}}\left( t,\,\psi ,\,x,\,u\right)= \Vert \psi \Vert +\Vert x\Vert +\Vert u\Vert .$$

${\mathcal {L}}$ satisfies all the conditions of hypothesis (H_L_). Then,

$${\mathcal {J}}(u)= \int _0^1\left( \Vert x_t^u\Vert +\Vert x^u(t)\Vert +\Vert u(t)\Vert \right) dt.$$

Hence, all the conditions of Theorem 11 are satisfied, and therefore, Eq. (21) has at least one optimal pair.

## Conclusions

In this work, we have considered a broader class of partial functional integrodifferential equations with finite delay in Banach spaces. Under some suitable conditions, we have shown the existence and uniqueness of mild solutions using contraction principle. Moreover, we showed the existence of optimal controls of the associated Lagrange problem using convex optimization techniques and Balder’s theorem. We also provided an example to illustrate our results which extend and complement many other important results in the literature.

## References

[CR1] Balder E (1987). Necessary and sufficient conditions for *L*_1_-strong–weak lower semicontinuity of integral functional. Nonlinear Anal Real World Appl.

[CR2] Boyarsky A (1976). On the existence of optimal controls for nonlinear systems. J Optim Theory Appl.

[CR3] Dafermos CM, Nohelj A (1979). Energy methods for non linear hyperbolic Volterra integrodifferential equations. Commun Partial Differ Equ.

[CR4] Ezzinbi K, Toure H, Zabsonre I (2009). Existence and regularity of solutions for some partial functional integrodifferential equations in Banach spaces. Nonlinear Anal.

[CR5] Flytzanis E, Papageorgiou NS (1991). On the existence of optimal controls for a class of nonlinear infinite dimensional systems. Math Nach.

[CR6] Grimmer R (1983). Resolvent operators for integral equations in a Banach space. Trans Am Math Soc.

[CR7] Grimmer R, Kappelf C (1984). Series expansions for resolvents of Volterra integrodifferential equations in Banach space. SIAM J Math Anal.

[CR8] Grimmer R, Pritchard AJ (1983). Analytic resolvent operators for integral equations in Banach space. J Differ Equ.

[CR9] Gurtin ME, Pipkin AC (1968). A genera1 theory of heat conduction with finite wave speeds. Arch Ration Mech Anal.

[CR10] Hongwei L (2003). Existence of optimal controls for semilinear parabolic equations without Cesari-type conditions. Appl Math Optim.

[CR11] Jakszto M, Skowron A (2003). Existence of optimal controls via continuous dependence on parameters. Comput Math Appl.

[CR12] Liang J, Liu JH, Xiao T-J (2008). Dynamics of continuous. Discrete Impuls Syst Ser A Math Anal.

[CR13] Li X, Liu Z (2015). The solvability and optimal controls of impulsive fractional semilinear differential equations. Taiwan J Math.

[CR14] Li X, Yong J (1995). Optimal control theory infinite dymensional systems.

[CR15] Lunardi A, Sinestrari E (1986). $$C^{\alpha }$$-regularity for non autonomous linear integrodifferential equations of parabolic type. J Differ Equ.

[CR16] Motta M, Rampazzo F (2013). Asymptotic controllability and optimal control. J Differ Equ.

[CR17] Noussair ES, Nababan S, Teo KL (1981). On the existence of optimal controls for quasilinear parabolic partial differential equations. J Optim Theory Appl.

[CR18] Pan X, Li X, Jing Z (2014). Solvability and optimal controls of semilinear Riemann–Liouville fractional differential equations. Abstr Appl Anal.

[CR19] Papageorgiou NS (1987). Existence of optimal controls for nonlinear systems in Banach spaces. J Optim Theory Appl.

[CR20] Sinestrari E, Boccardo L, Tesei A (1987). An integrodifferential equation arising from the theory of nonlinear heat flow with memory. Nonlinear parabolic equations: qualitative properties of solutions. Research notes in mathematics.

[CR21] Travis CC, Webb GF (1974). Existence and stability for partial functional differential equations. Trans Am Math Soc.

[CR22] Vrabie II (2003) *C*_0_-semigroups and applications. In: Lubkin S (eds) Nord-Holland Mathematics Studies, vol 191. Elsevier

[CR23] Wang J, Zhou Y, Medved M (2012). On the solvability and optimal controls of fractional integrodifferential evolution systems with infinite delay. J Optim Theory Appl.

[CR24] Wang JR, Zhou Y (2011). A class of fractional evolution equations and optimal controls. Nonlinear Anal Real World Appl.

[CR25] Wei W, Xiang X, Peng Y (2006). Nonlinear impulsive integrodifferential equation of mixed type and optimal controls. Optimization.

[CR26] Zhou J (2014). Optimal control of a stochastic delay partial differential equation with boundary-noise and boundary-control. J Dyn Control Syst.

